# Особенности водно-электролитного баланса у лиц старшей возрастной групп

**DOI:** 10.14341/probl13214

**Published:** 2024-01-24

**Authors:** Н. Н. Катамадзе, Е. А. Пигарова, Л. К. Дзеранова, Н. Г. Мокрышева

**Affiliations:** Национальный медицинский исследовательский центр эндокринологии; Национальный медицинский исследовательский центр эндокринологии; Национальный медицинский исследовательский центр эндокринологии; Национальный медицинский исследовательский центр эндокринологии

**Keywords:** водно-электролитный обмен, гипонатриемия, гипернатриемия, антидиуретический гормон, АДГ, ренин, альдостерон, РААС, старческий возраст, старение

## Abstract

Возрастные изменения оказывают большое влияние на регуляцию водно-электролитного гомеостаза в организме, который управляется сложным взаимодействием факторов окружающей среды, питьевым поведением, секрецией ряда гормонов и гормоноподобных веществ, а также иннервацией и функциональным состоянием почек. Хорошо известно, что изменения, которые являются частью физиологического старения, лежат в основе нарушений водно-электролитного баланса, что усугубляется наличием возраст-ассоциированных заболеваний, приемом лекарственных препаратов или рядом внешних факторов, таких как неполноценное питание, потребление жидкости, наличие деменции. В данном обзоре рассмотрены данные литературы по влиянию нормального старения на развитие патологии водно-натриевого баланса, включая дегидратацию пациентов старческого возраста, гипонатриемию, гипернатриемию, изменения секреции антидиуретического гормона и активности элементов ренин-ангиотензин-альдостероновой системы.

## Abstract (en)

Age-related changes have a great influence on the regulation of water and electrolyte homeostasis in the body, which is regulated by a complex interaction of environmental factors, drinking behavior, the secretion of a number of hormones and hormone-like substances, as well as the innervation and functional state of the kidneys. It is well known that the changes that are part of physiological aging underlie fluid and electrolyte imbalances, exacerbated by the presence of age-related diseases, medications, or a number of external factors such as malnutrition, fluid intake, and the presence of dementia. This review considers literature data on the effect of normal aging on the development of pathology of the water-sodium balance, including dehydration of senile patients, hyponatremia, hypernatremia, changes in the secretion of antidiuretic hormone and the activity of elements of the renin-angiotensin-aldosterone system.

## ВВЕДЕНИЕ

Нарушения водно-электролитного гомеостаза встречаются во всех областях клинической медицины и связаны со значительной заболеваемостью и смертностью, особенно среди лиц старше 75 лет [1]. По данным Роттердамского исследования из 776 человек старшей возрастной группы, 15% пациентов имели по крайней мере 1 нарушение электролитного баланса, при этом наиболее распространенными были гипонатриемия (7,7%) и гипернатриемия (3,4%) [2]. Согласно данным Всемирной организации здравоохранения (ВОЗ), к старшей возрастной группе относятся люди пожилого (60–74 года), старческого (75–90 лет) возрастов и долгожители (старше 90 лет). В связи с этим исследование механизмов возрастных изменений регуляции водно-электролитного баланса является актуальной проблемой.

Процесс старения неминуемо ведет к изменениям в системе гомеостаза, участвующей в регуляции водно-­электролитного баланса. В норме эта система функционирует для поддержания состава жидкости и электролитов в пределах узкого референсного диапазона. Важными механизмами поддержания данного гомеостаза в организме человека являются взаимовлияние гормонов нейроэндокринной системы (антидиуретический гормон (АДГ, син. вазопрессин), апелин), ряда натрийуретических пептидов, гормонов надпочечников (альдостерон), а также восприятие чувства жажды, которое регулирует потребление жидкости, работу мочевыделительной системы, дополнительно координируемой еще и нейрональными влияниями.

Показано, что изменения, которые являются частью нормального старения, лежат в основе нарушений водно-электролитного баланса, что усугубляется наличием возраст-ассоциированных заболеваний, приемом лекарственных препаратов или рядом внешних факторов, таких как неполноценное питание и потребление жидкости.

В данном обзоре основное внимание уделено возрастным изменениям, влияющим на водно-электролитный гомеостаз, и клиническим последствиям, к которым эти изменения могут приводить.

## ОБЕЗВОЖИВАНИЕ У ПОЖИЛЫХ ЛЮДЕЙ

Люди старческого возраста очень восприимчивы к обезвоживанию, которое является наиболее частой причиной почечной недостаточности и водно-электролитных нарушений [1]. Триггером обезвоживания может служить воздействие даже легких стрессов, таких как ограничение воды, лихорадка, инфекционное заболевание или диарея. Поскольку прием жидкости — единственный способ пополнить дефицит воды, а почки играют первостепенную роль в снижении дальнейших потерь жидкости, снижение чувства жажды и ухудшение концентрационной способности почек играют важную роль в предрасположенности пожилых людей к обезвоживанию.

Жажда может рассматриваться как защитный механизм организма в ответ на предполагаемый дефицит жидкости. Считается, что осморецепторные клетки локализованы в сосудистом органе терминальной пластинки и участках прилежащего переднего гипоталамуса вблизи передней стенки третьего желудочка мозга [3]. Когда эти клетки обезвожены, они задействуют нейронные цепи и активируют центральные системы секреции АДГ и жажды.

АДГ секретируется в крупноклеточных нейронах супраоптического и паравентрикулярного ядер гипоталамуса в ответ на активацию осморецепторов. Классические биологические функции АДГ осуществляет благодаря связыванию с вазопрессиновыми рецепторами 2 типа (AVP2R) на базолатеральной мембране главных клеток собирательных трубочек. АДГ увеличивает проницаемость для воды в собирательных трубочках, тем самым обеспечивая осмотическое равновесие между мочой и гипертоническим мозговым интерстицием. Конечным результатом этого процесса является повышение концентрации мочи и уменьшение ее объема (антидиурез) [4][5].

## СИСТЕМА РЕГУЛЯЦИИ ВОДНО-ЭЛЕКТРОЛИТНОГО ОБМЕНА

Активация осморецепторов гипоталамической области, которая происходит при повышении осмотического давления крови более 280 мосм/л Н2О, а также волюморецепторов левого предсердия при уменьшении объема крови усиливает синтез АДГ cупраоптическим и паравентрикулярным ядрами гипоталамуса. АДГ, в свою очередь, усиливает реабсорбцию воды в канальцах нефронов [6].

Ишемия почек, активация рецепторов приводящих артериол нефрона при уменьшении почечного кровотока или кровопотере, а также натриевых рецепторов плотного пятна юкстагломерулярного комплекса при дефиците натрия усиливают синтез и высвобождение в кровь ренина и его активность. Образующийся под влиянием ренина ангиотензин II активно воздействует на почечную гемодинамику.

Ангиотензин II, активируя ангиотензиновые рецепторы 2 типа (AT2), действует как сосудосуживающее средство в междольковых артериях, а также в приносящей и выносящей клубочковых артериолах, с большим эффектом на выносящую. Эти действия повышают внутриклубочковое капиллярное давление. Преимущественная вазоконстрикция выносящей артериолы и повышение гидростатического давления в клубочках помогают сохранять скорость клубочковой фильтрации (СКФ) в состоянии активации ренин-ангиотензин-альдостероновой системы, но мезангиальная, афферентная и системная вазоконстрикция снижают почечный кровоток и, как следствие, СКФ. Конечным результатом действия ангиотензина II является увеличение фильтрационной способности почек на фоне относительно небольшого снижения СКФ [7].

Не менее важным является действие ангиотензина II в канальцевом аппарате почек. Порядка 60–80% воды и растворенных веществ, отфильтрованных в клубочках, реабсорбируется в проксимальных канальцах, а ангио­тензин II оказывает прямое влияние на этот процесс. Принято считать, что ангиотензин II стимулирует транспорт натрия и воды в проксимальных канальцах почек [8]. Реабсорбция натрия (Na) и бикарбоната (HCO3–) осуществляется за счет люминального Na+/H+ (NHE3) и базолатерального Na+/HCO3– котранспортеров. Субнаномолярные концентрации ангиотензина II стимулируют оба этих обменника, что приводит к увеличению транспорта натрия и воды, а также к повышенной реабсорбции HCO3. Кроме того, ангиотензин II стимулирует экспрессию H+/-АТФазы, Na+/K+-АТФазы и активирует котранспортер Na+/глюкозы, что приводит к увеличению абсорбции натрия, воды и бикарбоната [9][10].

Следует помнить, что основная часть опосредованных ренин-ангиотензин-альдостероновой системой (РААС) эффектов связана с действием альдостерона в дистальных извитых канальцах. Альдостерон стимулирует реабсорбцию натрия в дистальных отделах за счет своего воздействия как на cимпортер хлорида натрия, также известный как Na+/Cl–-котранспортер (NCC), так и на эпителиальные натриевые каналы собирательных трубочек (ENaC) [11].

Уменьшение объема внеклеточной жидкости и ангиотензин II повышают активность центра жажды в гипоталамусе. Антидиуретическим и антинатрийуретическим механизмам противостоят диуретические и натрий­уретические. Главными действующими факторами их являются реномедуллярные почечные простагландины и атриопептиды [11].

Диуретический эффект опосредован снижением высвобождения АДГ с одновременным увеличением высвобождения предсердного натрийуретического пептида (ПНП). ПНП вырабатывается в клетках предсердий, он подавляет реабсорбцию натрия, повышает СКФ, снижает проницаемость собирательных трубочек почек для воды, способствуя диурезу и натрийурезу, ингибирует вазопрессорный ответ на ангиотензин II и эндотелин-1, снижает системное артериальное давление и частоту сердечных сокращений, секрецию АДГ, альдостерона и ренина [12]. Содержание ПНП в предсердиях и секреция его в кровь увеличиваются под влиянием приема избыточного количества воды и поваренной соли, растяжения предсердий, при повышении ­артериального ­давления, а также при стимуляции рецепторов AVP2R и α-адренорецепторов [12].

Свои функции ПНП осуществляет посредством сложных молекулярных механизмов. В почках ПНП связывается с рецептором натрийуретического пептида-А (NPR-A) и усиливает его гуанилатциклазную активность, тем самым увеличивая внутриклеточную выработку циклического гуанозинмонофосфата, способствуя натрий­урезу. Противодействуя эффектам РААС, в клубочковых капиллярах под влиянием ПНП увеличивается скорость фильтрации. По ходу нефрона натрийуретическое и диуретическое действие ПНП опосредовано ингибированием базолатеральной Na+/K+-АТФазы, снижением апикального переносчика натрия, калия и белковых органических катионов в проксимальных канальцах — Na+/K+/Cl–-котранспортера. В собирательных трубочках ПНП снижает реабсорбцию натрия путем ингибирования катионных каналов, управляемых циклическими нуклеотидами, эпителиального натриевого канала и гетеромерного канала [12].

Данные механизмы функционируют постоянно и обеспечивают восстановление водно-электролитного гомео­стаза при кровопотере и обезвоживании, избытке воды в организме, а также при колебаниях осмотической концентрации внеклеточной жидкости. Изменения водного обмена обозначаются как положительный (накопление в организме избытка воды) или отрицательный (дефицит в организме воды) водный баланс. Если эффективность системы регуляции водного баланса недостаточна, развиваются различные варианты нарушений.

## ИЗМЕНЕНИЯ ФУНКЦИОНИРОВАНИЯ ПОЧЕК ПРИ НОРМАЛЬНОМ СТАРЕНИИ

Нормальное старение сопровождается изменениями анатомии и функции почек (табл. 1). Почечная масса постепенно снижается от своего первоначального веса примерно 250–340 г у молодых людей до 180–200 г к возрасту 80–90 лет [13]. С возрастом количество гистологически здоровых клубочков уменьшается, а процент гиалинизированных и склерозированных — увеличивается [14]. Таким образом, происходит уменьшение эффективной фильтрующей поверхности почек, увеличение количества мезангиальных клеток, снижение количества эпителиальных клеток и утолщение базальной мембраны клубочков.

**Table:** Таблица 1. Влияние старения на регуляцию натрия и воды

Возрастные изменения в почках	•Снижение массы почек. •Снижение почечного кровотока. •Снижение СКФ. •Нарушение разбавляющей способности дистальных почечных канальцев. •Снижение концентрационной способности почек. •Нарушение сохранения уровня натрия. •Развитие резистентности почек к действию АДГ
Гормональные изменения	•АДГ. •Нормальная или повышенная базальная секреция. •Повышенная реакция на осмотическую стимуляцию. •Снижение ночной секреции. •РААС. •Снижение активности ренина плазмы. •Снижение продукции альдостерона
Потребление жидкости	•Снижение потребления жидкости. •Снижение восприятия чувства жажды

В рамках нормального процесса старения наблюдаются изменения в гемодинамике почек: сужается просвет междолевых и дуговых артерий, повышается извитость внутридольковых капилляров. В кортикальном слое почек происходит отложение гиалина в стенках артериол, что приводит к атрофии гладкомышечных клеток, облитерации просвета артериол и утрате клубочковых капилляров. В юкстамедуллярной области гломерулярный склероз может приводить к появлению анастомозов между афферентными и эфферентными артериолами. Однако приток крови к мозговому веществу почек сохраняется через прямые артерии и в старческом возрасте [15].

Анатомические изменения стареющей почки сопровождаются изменениями функции почек, несмотря на то, что прямая связь между анатомическими и функциональными изменениями не установлена, известно, что почечный кровоток снижается в ходе процесса старения примерно на 10% за каждое десятилетие после достижения юношеского возраста, так что к 90 годам почечный кровоток составляет примерно 300 мл/мин, что на 50% меньше, чем в 30-летнем возрасте. Снижение почечной перфузии наиболее выражено в корковом веществе почек с минимальным влиянием на мозговое вещество [16].

СКФ остается относительно стабильной до 30 лет, после чего снижается примерно на 0,7–0,9 мл/мин/1,73 м² в год. Последствием старения также является снижение не только выделительной, но и концентрационной функции, а также натрий-сберегающей способности почек [17]. Эти изменения снижают способность почек сохранять натрий и воду в условиях повышенных потерь и могут приводить к гиповолемии и обезвоживанию. С другой стороны, неспособность выделять избыточную жидкость приводит к тому, что люди старческого возраста подвержены риску водной интоксикации и гипонатриемии.

## РОЛЬ ПОЧЕК В ПОДДЕРЖАНИИ ВОДНО-ЭЛЕКТРОЛИТНОГО БАЛАНСА

Реабсорбция воды из собирательных трубочек осуществляется благодаря созданию градиента концентрации (противотока) между интерстицием и канальцевым аппаратом почек. Градиент концентраций создается за счет накопления мочевины и ионов натрия в интерстициальной жидкости и сосудах почек (vasa recta). Формирование конечной мочи — довольно сложный процесс. Канальцевая моча сначала осмотически концентрируется в нисходящем отделе петли Генле, затем разбавляется в восходящем отделе петли Генле, прежде чем окончательно сконцентрироваться в собирательных трубочках [18] .

Когда два раствора разделены мембраной, которая проницаема для воды и непроницаема для растворенных веществ, вода перемещается через мембрану от раствора с меньшей концентрацией к раствору с большей концентрацией, пока оба раствора не достигнут равновесия. Это движение называется осмосом. Осмос не может продолжаться бесконечно — он прекращается, когда растворенные вещества по обе стороны мембраны имеют одинаковую осмотическую силу (осмотическое давление). Осмоляльность мочи в собирательных трубочках составляет приблизительно 60 мОсм/л, тогда как осмоляльность интерстициальной жидкости — приблизительно 1200 мОсм/л. АДГ приводит к активации каскада реакций с вовлечением цАМФ, что ведет к движению пузырьков с аквапорином-2 (AQP-2), являющимся транспортным белком для воды клеточной мембраны, и встраиванию этих пузырьков в апикальную мембрану клеток, обращенную в сторону первичной мочи, что способствует увеличению проницаемости собирательных трубочек для воды, натрия и мочевины, тем самым обеспечивая осмотическое равновесие между канальцевой мочой и интерстицием почек. Конечным результатом этого процесса является извлечение воды из канальцевой мочи, что приводит к повышению ее концентрации и уменьшению объема (антидиурез). Самым активным осмолем в собирательных трубочках является мочевина, создающая концентрационный градиент в медуллярном интерстиции почек для обеспечения антидиуретического действия в собирательных трубочках. Важность роли мочевины в механизме концентрации мочи была подтверждена дефектами концентрации мочи в различных мышиных моделях с нокаутированными переносчиками мочевины [19].

АДГ увеличивает фосфорилирование и накопление транспортеров мочевины (UT-A1, UT-A3) в апикальной мембране и тем самым активирует эти транспортеры. Важно отметить, что собирательные трубочки мозгового слоя почек являются единственным участком, в котором под действием АДГ увеличивается проницаемость для мочевины, а не для воды, как в остальных частях собирательных трубочек, тем самым повышается концентрационный градиент [20].

Иннервация почек также вносит большой вклад в поддержание осмоляльности крови. Осмоляльность внеклеточной жидкости практически всецело принадлежит натрию, поэтому даже минимальные изменения его концентрации приводят к существенным электролитно-метаболическим сдвигам внутри клетки, перемещению воды через клеточную мембрану в сторону градиента концентраций и изменению объема клеток. Почки интенсивно иннервированы симпатическими нейронами, которые способны напрямую повышать канальцевую реабсорбцию натрия. Симпатическая активация также может повышать секрецию ренина и кровоснабжение почек, что косвенно способствует реабсорбции натрия. Было показано, что активация дофаминовых рецепторов, обнаруживаемых по ходу почечных трубочек и почечных сосудов, приводит к натрийурезу, диурезу и расширению почечных сосудов [21].

В почечном клубочке ангиотензин II, норадреналин и эндотелин приводят к сокращению артериол, тогда как простагландины I и E, брадикинин, ANP и дофамин оказывают сосудорасширяющее действие. В почечных трубочках ангиотензин II, норадреналин, гормон роста и инсулин стимулируют реабсорбцию натрия, тогда как дофамин и паратиреоидный гормон ее подавляют. В петле Генле катехоламины стимулируют реабсорбцию натрия, тогда как простагландин Е способствует натрийурезу [21].

## ВОЗРАСТНЫЕ ИЗМЕНЕНИЯ РЕГУЛЯЦИИ ВОДНО-ЭЛЕКТРОЛИТНОГО ОБМЕНА У ПОЖИЛЫХ ПАЦИЕНТОВ

## Нарушение выведения воды почками в пожилом возрасте

Способность выводить воду из организма определяется такими факторами, как адекватная доставка растворенного вещества в область разведения (почечная перфузия и СКФ), функционально интактный дистальный участок разведения (дистальный каналец и восходящая часть петли Генле) и подавление АДГ во избежание реабсорбции воды в собирательных трубочках [22]. Почки пожилых людей в меньшей степени способны разбавлять мочу и выводить воду. Наиболее важным фактором в данном процессе является возрастное снижение СКФ [17].

Согласно литературным данным, у пожилых людей высвобождение эндогенного АДГ в ответ на тесты с осмотической стимуляцией, например инфузию гипертонического раствора или 24-часовую депривацию воды, является аномально высоким, что свидетельствует о повышенной чувствительности осморецепторов гипоталамуса к повышению осмоляльности крови [23][24]. Обычно повышение осмоляльности плазмы всего на 1–2% является достаточным для увеличения концентрации АДГ в плазме до 1 пг/мл. Следует отметить, что существует осмотический порог высвобождения АДГ для любого человека, и этот чувствительный механизм помогает поддерживать осмоляльность плазмы в диапазоне от 275 до 295 мОсм/кг H2O. АДГ взаимодействует с собственным рецептором AVP2R, который экспрессируется в собирательных трубочках почек.

Возрастных дегенеративных изменений ядер, в которых продуцируется АДГ, по-видимому, не наблюдается. Нет никаких признаков разрушения клеток, уменьшения количества нейронов или потери разветвления дендритов, что часто обнаруживается в других отделах стареющего мозга. Более того, количество нейросекреторного материала в данных ядрах не отличается по количеству от такового у более молодых субъектов [25]. С другой стороны, у пожилых людей снижена чувствительность почечных канальцев к действию АДГ. Исследование, проведенное Catudioc-Vallero J. и соавт. на крысах в возрасте 8–9 мес, показало, что хроническое воздействие на почки повышенного содержания АДГ приводит к снижению чувствительности почек к гормону, возможно, за счет подавления экспрессии AVP2R и передачи внутриклеточных сигналов [26].

## Изменение баланса натрия в старческом возрасте

В норме стареющая почка без приобретенного заболевания способна адекватно регулировать уровень натрия в крови. Возраст-ассоциированное снижение почечного кровотока и СКФ способствует пассивной реабсорбции жидкости, что увеличивает риск водной интоксикации и гипонатриемии. В целом способность стареющей почки эффективно реабсорбировать натрий снижена. Факторы, связанные с этим явлением, включают влияние возраста на снижение количества функционирующих нефронов, уровня ренина, альдостерона [27].

У пациентов старческого возраста реабсорбция натрия в проксимальных канальцах соответствует таковой в молодом возрасте, но заметно снижена способность к реабсорбции натрия в восходящем отделе петли Генле. Это приводит к увеличению количества натрия, доставляемого в дистальные сегменты, и к снижению способности создания градиента концентрации в интерстиции мозгового вещества. Результатом данных изменений является снижение способности концентрировать мочу в пожилом возрасте [28].

## Возрастные изменения в РААС

Активность РААС существенно изменяется с возрастом, что обусловлено не только фактором старения, но и применением большого количества лекарственных средств, оказывающих непосредственное влияние на различные звенья данной системы.

A. Fernández-Atucha и соавт. [29] обнаружили, что у пожилых мужчин активность ангиотензинпревращающего фермента в сыворотке крови ниже, чем у молодых мужчин. Доказано, что здоровые пожилые люди в возрасте 62–70 лет имеют более низкую концентрацию альдостерона и активность ренина в плазме [27]. Снижение концентрации альдостерона, по-видимому, является прямым результатом снижения активности ренина плазмы, а не возрастных изменений в надпочечниках, поскольку синтез альдостерона и кортизола на фоне инфузии адренокортикотропного гормона у пожилых людей остается неизменным.

## КЛИНИЧЕСКИ ЗНАЧИМЫЕ НАРУШЕНИЯ ВОДНО-ЭЛЕКТРОЛИТНОГО ОБМЕНА У ЛЮДЕЙ СТАРЧЕСКОГО ВОЗРАСТА

Наиболее распространенными и клинически значимыми нарушениями водно-электролитного обмена у людей старческого возраста являются гипонатриемия и гипернатриемия.

## Гипонатриемия

Гипонатриемия, определяемая как уровень Na в сыворотке <135 ммоль/л, является наиболее частым нарушением электролитного баланса у людей в старческом возрасте [30]. M. Miller и соавт., исследовавшие 405 амбулаторных пациентов старческого возраста, средний возраст которых составил 78 лет, сообщили о 11% случаев гипонатриемии [31]. В другом наблюдательном исследовании этих же авторов [32] обнаружено, что частота гипонатриемии была в 2 раза выше и составила порядка 22% среди пациентов старческого возраста, проживающих в учреждениях длительного ухода. В литературе также представлены данные о том, что среди госпитализированных пациентов старческого возраста гипонатриемия может возникать еще чаще. В исследовании M.Y. Rao и соавт. показано, что у 518 (36%) из 1440 пациентов старше 74 лет, поступивших в отделение интенсивной терапии в течение 18 мес, уровень натрия в сыворотке крови был <135 ммоль/л, а у 100 (6,9%) пациентов — <125 ммоль/л [33].

Гипонатриемия отражает дисбаланс между уровнями воды и натрия в плазме. Риск развития гипонатриемии значительно выше у пациентов старческого возраста из-за нарушения физиологической регуляции водно-­электролитного баланса, повышенной распространенности заболеваний, вызывающих гипонатриемию, и полипрагмазии [34].

Факторы риска гипонатриемии делятся на следующие категории.

Ограниченная диета по потреблению натрия у лиц старшего возраста (например, диета «тосты и чай») может сама по себе приводить к развитию гипонатриемии. Этот тип гипонатриемии может возникнуть у людей старческого возраста со сниженной СКФ, которые придерживаются диеты с низким содержанием соли и белка, но при этом пьют большое количество воды. В этих случаях наблюдаются уменьшение фильтрата в канальцевом аппарате почек из-за сниженной СКФ и, возможно, хронического абсолютного дефицита натрия, а также повышение реабсорбции воды в связи с ограниченной скоростью экскреции осмолей (Na, мочевины); таким образом, когда потребление воды превышает способность почек экскретировать воду, возникает гипонатриемия [35].

Состояния, связанные со снижением способности выводить воду у пациентов старческих возрастных групп, представлены в таблице 2.

**Table:** Таблица 2. Состояния, связанные со снижением выделения жидкости из организма

1.Возрастное снижение способности почек экскретировать разбавленную мочу
2.Идиопатический синдром неадекватной секреции антидиуретического гормона (СНCАДГ)
3.Лекарственно-индуцированный СНCАДГ
4.Воспалительное заболевание легких с СНСАДГ
5.Злокачественное новообразование с СНCАДГ
6.Поражение ЦНС

Согласно литературным данным, среди людей старческого возраста СНСАДГ является наиболее частой причиной гипонатриемии, а от 26 до 60% случаев СНСАДГ имеют идиопатическую природу [36].

Гипонатриемия у людей старшей возрастной группы связана со снижением нейрокогнитивных функций, нестабильностью походки, падениями, частыми переломами костей, развитием остеопороза, частотой повторных госпитализаций, потребностью в длительном уходе и высокой смертностью [37][38].

Тяжелая/выраженная гипонатриемия (менее 125 ммоль/л) может привести к смерти вследствие вклинения миндалин мозжечка в большое затылочное отверстие, вызванного отеком головного мозга. В норме церебральная атрофия защищает людей старческого возраста от развития такого вклинения, поскольку в черепе остается «больше места». Однако наличие внутричерепного кровоизлияния или опухоли может подвергать пациентов старших возрастных групп высокому риску развития отека головного мозга. Кроме того, гипоксия, наиболее важный фактор риска смерти у пациентов с выраженной гипонатриемией, нередко встречается у данной группы пациентов. Легкая гипонатриемия может вызвать анорексию, а пациенты с гипонатриемией в гериатрическом отделении демонстрируют значительно более низкую приверженность к питанию, чем пациенты с эунатриемией [39].

B. Renneboog и соавт. обнаружили, что пациенты с легкой/умеренной хронической «асимптоматической» гипонатриемией имеют низкие показатели при оценке когнитивных функций, таких как повседневная активность, тест на оценку психического состояния, шкала гериатрической депрессии, тест на подвижность и т.д., по сравнению с пациентами, у которых отмечалась эунатриемия. В этом же исследовании показано, что у «бессимптомных» пациентов с гипонатриемией вероятность падения была в 67 раз выше, чем у пациентов с нормонатриемией. Показательным является также то, что нестабильность походки улучшалась при коррекции гипонатриемии [40][41].

Несколько исследований показали повышенную частоту переломов у пациентов с гипонатриемией, следовательно, можно рассмотреть вопрос целесообразности оценки уровня натрия в сыворотке крови у людей старческого возраста с переломами и/или неустойчивой походкой [42]. J.G. Verbalis и соавт. продемонстрировали на крысиной модели, как хроническая гипонатриемия может приводить к увеличению количества остеокластов и потере костной массы [43]. По данным американской национальной программы социального исследования (NHANES III), умеренная гипонатриемия у пациентов старческого возраста связана с риском развития остеопороза. Риск переломов повышен у пациентов с гипонатрие­мией [44] из-за высокой частоты спонтанных падений и, возможно, из-за вызванного гипонатриемией остеопороза. Согласно данным R. Tolouian и соавт., у пациентов с переломом шейки бедра после случайного падения вероятность наличия гипонатриемии в 4,8 раза выше по сравнению с контрольной группой [45]. Эти данные, безусловно, подтверждают высокую социальную значимость диснатриемии в пожилом и старческом возрасте.

Гипонатриемия может быть разделена на следующие категории: эуволемическая, гиповолемическая, гиперволемическая.

Как указывалось ранее, СНСАДГ является наиболее частой причиной гипонатриемии у людей старческого возраста и по определению связан с эуволемической гипонатриемией. Гипотиреоз редко вызывает гипонатриемию, но в тяжелых случаях, таких как микседема, может привести к снижению сердечного выброса и СКФ, что повышает секрецию вазопрессина и нарушает возможности почек выводить свободную воду [46].

Гиперволемическую гипонатриемию можно наблюдать у пациентов с застойной сердечной недостаточностью, циррозом печени и почечной недостаточностью, а также при приеме избыточного количества воды [46].

Гиповолемическая гипонатриемия может быть вызвана плохо контролируемым или невыявленным сахарным диабетом, первичной надпочечниковой недостаточностью, синдромом церебральной потери соли, диареей, рвотой и применением диуретиков. При вторичной надпочечниковой недостаточности снижение уровня кортизола приводит к отсутствию подавления вазопрессина с последующим нарушением экскреции свободной воды, тогда как при первичной надпочечниковой недостаточности снижение уровня альдостерона приводит к потере натрия почками [47].

Прием тиазидных диуретиков является ведущей причиной гиповолемической гипонатриемии. Гипонатриемия, индуцированная тиазидами, представляет собой серьезный побочный эффект лечения этими препаратами, и наблюдается очень часто — до 1 случая на 7 пациентов, получающих тиазидные диуретики [48]. Препараты данной группы усиливают диурез, нарушая реабсорбцию натрия за счет блокады Na+/Cl--котранспортера (SLC12A3) в проксимальном сегменте дистального извитого канальца. Когда канал Na+/Cl- заблокирован, сниженные уровни натрия, проникающего через люминальную мембрану, угнетают действие Na+/K+-насоса, уменьшая прохождение Na и воды в интерстиций [49]. Cокращение внеклеточного объема жидкости стимулирует барорегулируемую секрецию АДГ, которая приводит к максимальной реабсорбции воды, что требует существенной потери натрия и массы тела. Было бы разумно предположить, что тиазиды непосредственно индуцируют высвобождение АДГ или усиливают реакцию собирательных трубочек на циркулирующий АДГ. По-видимому, существует индивидуальная восприимчивость к этим эффектам, поскольку гипонатриемия возникает не у всех пациентов и обычно рецидивирует при повторном введении препаратов.

В большинстве случаев гипонатриемия развивается вскоре после начала приема препарата, хотя может произойти и на более позднем этапе лечения. Этот побочный эффект может сохраняться в течение месяца после прекращения приема препарата, но, как правило, быстро регрессирует после прекращения приема тиазидных диуретиков [48].

Гипонатриемия может возникать также вследствие прямых почечных потерь натрия — с неадекватным натрийурезом. Развившаяся в результате этого гиповолемия стимулирует соответствующее неосмотическое высвобождение вазопрессина, что приводит к снижению выделения воды почками, но не может компенсировать потерянный объем жидкости и натрия с мочой. Это может быть следствием заболеваний почек, таких как кистозная болезнь почек, анальгетическая нефропатия, хронический пиелонефрит и обструктивная уропатия.

Лечение гипонатриемии

Лечение людей старших возрастных групп с гипонатриемией должно быть направлено на устранение основной причины с учетом остроты, тяжести и степени гипонатриемии у соответствующего пациента. Введение внутривенного физиологического раствора (0,9%) обычно является основным методом лечения гиповолемической гипонатриемии.

У пациентов с эуволемической гипонатриемией тактикой лечения может являться ограничение жидкости в зависимости от тяжести симптомов, остроты и степени гипонатриемии. Цель лечения гипонатриемии — выделение мочи на 500 мл больше, чем прием жидкости внутрь. Однако это относительно сложно реализовать на практике. Предикторами вероятной неэффективности ограничения жидкости являются высокая осмоляльность мочи (>500 мОсм/кг H2O), объем суточной мочи менее 1500 мл в сутки, снижение концентрации натрия в сыворотке менее 2 ммоль/л/сут в течение 24–48 ч ограничения жидкости менее 1000 мл/сут [50].

При неэффективности ограничения жидкости используют введение 3% гипертонического раствора. У лиц с тяжелыми симптомами и/или концентрацией Na в сыворотке <120 ммоль/л рекомендуется быстрое болюсное вливание 100–150 мл гипертонического 3% раствора в течение 10–20 мин, что обычно приводит к повышению уровня натрия в сыворотке крови более чем на 3–4 ммоль/л. Повышение сывороточного Na от 4 до 8 ммоль/л и не более 10 ммоль/л в течение первых 24 ч или у пациентов с высоким риском синдрома осмотической демиелинизации от 4 до 6 ммоль/л в день является безопасным для пациента. Есть также данные о введении гипертонического раствора вместе с десмопрессином или коррекции чрезмерного (выше желаемого) содержания натрия в крови с помощью десмопрессина с одновременным введением 5% декстрозы, растворенной в воде, что применяется для управления скоростью коррекции натрия в крови [51].

## Гипернатриемия

Распространенность гипернатриемии заметно увеличивается с возрастом. Основной физиологической реакцией организма на гипернатриемию является усиление жажды. Однако у людей старческого возраста восприятие жажды снижено. Это может быть связано с ослаблением когнитивных функций, приемом различных лекарственных препаратов или возрастным поражением гипоталамуса, например, вследствие поражения мелких сосудов. Происходит также снижение секреции ангиотензина II, что играет важную роль в развитии гипернатриемии [27]. Кроме того, способность почек выводить натрий значительно уменьшается из-за сниженной чувствительности к вазопрессину на уровне канальцевых рецепторов и снижения концентрационного градиента в мозговом веществе почек [52].

N.A. Snyder и соавт. проанализировали данные 120 и 137 госпитализированных в стационар пациентов и обнаружили, что уровень натрия в сыворотке >145 ммоль/л отмечался у 1,5% пациентов, получавших неотложную помощь в стационаре, и у 0,6% пациентов, получавших плановое стационарное лечение. В другом ретроспективном исследовании, включавшем 15 187 пациентов, 162 (1,1%) пациента были старше 60 лет, при этом у 57% развилась гипернатриемия во время госпитализации, а у остальных 43% — гипернатриемия была зарегистрирована при поступлении [53].

К факторам риска развития гипернатриемии относят повышенную потерю воды и снижение ее потребления. Развитие гипернатриемии вследствие повышенного потребления соли в целом является казуистикой. Этиология повышенной потери воды включает: осмотический диурез (вызванный диуретиками или глюкозурией), некомпенсированный несахарный диабет (абсолютный дефицит или резистентность к вазопрессину) [54], возрастное нарушение концентрационной способности почек, резистентность к действию вазопрессина (возрастная и приобретенная (например, лекарственные препараты)), заболевание почечных канальцев, осмотическая диарея, рвота, неощутимая потеря жидкости с чрезмерным потоотделением или тахипноэ.

Причины снижения потребления воды включают нарушение восприятия жажды и когнитивных функций у людей старческого возраста или инвалидизацию пациентов, обеспечивающую доступ к жидкости. Неадекватное потребление жидкости, как правило, наблюдается у ослабленных людей или вторично по отношению к инсульту, депрессии или слабоумию [53].

Гипернатриемия у людей старческого возраста может быть бессимптомной, поскольку классические симптомы обезвоживания, такие как жажда, часто отсутствуют [55]. Клинические проявления гипернатриемии включают снижение тургора кожи, сухость слизистых оболочек полости рта и изменение сознания.

Смертность от гипернатриемии у пациентов старческого возраста довольно высока, увеличиваясь с повышением тяжести гипернатриемии. Снижение когнитивных функций при поступлении является основным прогностическим фактором смертности пациентов [56]. Гипернатриемию следует корректировать увеличением потребления жидкости. В зависимости от самочувствия пациента, факта наличия/отсутствия у него сахарного диабета может потребоваться внутривенное введение 5% раствора декстрозы или энтеральное введение воды. Однако смертность тем выше, чем больше скорость замещения жидкости. Поэтому, как правило, рекомендуется постепенно корректировать гипернатриемию в течение не менее 48 ч со скоростью, не превышающей 12 ммоль/л/день [56]. Главной же задачей является устранение основных причин возникновения гипернатриемии с целью предотвращения рецидива. В некоторых случаях, например при очень тяжелой деменции, это может оказаться невозможным, и следует рассмотреть паллиативный подход в ведении пациента.

## ЗАКЛЮЧЕНИЕ

Водно-электролитные нарушения среди лиц старшей возрастной группы в клинической практике встречаются чаще, чем у молодых. Понимание физиологических и патологических механизмов, лежащих в основе предрасположенности к водно-электролитным нарушениям, может помочь клиницистам свести до минимума связанные с данными состояниями заболеваемость и смертность.

## ДОПОЛНИТЕЛЬНАЯ ИНФОРМАЦИЯ

Источник финансирования. Работа выполнена по инициативе авторов без привлечения финансирования.

Конфликт интересов. Авторы декларируют отсутствие явных и потенциальных конфликтов интересов, связанных с публикацией настоящей статьи.

Участие авторов. Все авторы одобрили финальную версию статьи перед публикацией, выразили согласие нести ответственность за все аспекты работы, подразумевающую надлежащее изучение и решение вопросов, связанных с точностью или добросовестностью любой части работы.
